# RETRACTION: Chemosensitization in Non‐small Cell Lung Cancer Cells by IKK Inhibitor Occurs via NF‐κB and Mitochondrial Cytochrome C Cascade

**DOI:** 10.1111/jcmm.70160

**Published:** 2024-10-22

## Abstract

**RETRACTION**: X. Jin, L. Qiu, D. Zhang, M. Zhang, Z. Wang, Z. Guo, C. Deng, and C. Guo, “Chemosensitization in Non‐small Cell Lung Cancer Cells by IKK Inhibitor Occurs via NF‐κB and Mitochondrial Cytochrome C Cascade,” *Journal of Cellular and Molecular Medicine* 13, no. 11–12 (2009): 4596–4607, https://doi.org/10.1111/j.1582‐4934.2008.00601.x.

The above article, published online on 4 March 2010 in Wiley Online Library (wileyonlinelibrary.com), has been retracted by agreement between the journal Editor‐in‐Chief, Stefan N. Constantinescu; the Foundation for Cellular and Molecular Medicine; and John Wiley & Sons Ltd.

The retraction has been agreed due to concerns raised by third parties on the data presented in the article. Specifically, image elements in Figures 2A and 6 were found to have been previously published by the same author group in a different scientific context. Further investigation by the publisher uncovered that the same concerns affected additional image elements in Figures 3B, 3C and 7A. The corresponding author was unresponsive to requests for clarification. For these reasons, the article is retracted as its conclusions are deemed invalid by the editors. Confirmation of retraction could not be obtained by the remaining co‐authors.

**RETRACTION**: X. Jin, L. Qiu, D. Zhang, M. Zhang, Z. Wang, Z. Guo, C. Deng, and C. Guo, “Chemosensitization in Non‐small Cell Lung Cancer Cells by IKK Inhibitor Occurs via NF‐κB and Mitochondrial Cytochrome C Cascade,” *Journal of Cellular and Molecular Medicine* 13, no. 11–12 (2009): 4596–4607, https://doi.org/10.1111/j.1582‐4934.2008.00601.x.

The above article, published online on 4 March 2010 in Wiley Online Library (wileyonlinelibrary.com), has been retracted by agreement between the journal Editor‐in‐Chief, Stefan N. Constantinescu; the Foundation for Cellular and Molecular Medicine; and John Wiley & Sons Ltd.

The retraction has been agreed due to concerns raised by third parties on the data presented in the article. Specifically, image elements in Figures 2A and 6 were found to have been previously published by the same author group in a different scientific context. Further investigation by the publisher uncovered that the same concerns affected additional image elements in Figures 3B, 3C and 7A. The corresponding author was unresponsive to requests for clarification. For these reasons, the article is retracted as its conclusions are deemed invalid by the editors. Confirmation of retraction could not be obtained by the remaining co‐authors.

